# A qualitative exploration of smokers' views regarding aspects of a community-based mobile stop smoking service in the United Kingdom

**DOI:** 10.1186/1471-2458-11-873

**Published:** 2011-11-16

**Authors:** Manpreet Bains, Andrea Venn, Rachael L Murray, Ann McNeill, Laura L Jones

**Affiliations:** 1 UK Centre for Tobacco Control Studies, Division of Epidemiology and Public Health, University of Nottingham, Nottingham, UK

## Abstract

**Background:**

Developing more accessible stop smoking services (SSS) is important, particularly for reaching smokers from socio-economically deprived groups who are more likely to smoke and less likely to quit in comparison to their more affluent counterparts. A drop-in mobile SSS (MSSS) was piloted across 13 locations in socio-economically deprived areas of Nottingham.

**Methods:**

Semi-structured telephone interviews were conducted to explore the views of 40 smokers who registered with the MSSS.

**Results:**

The MSSS appeared to trigger quit attempts. For some of the participants the attempt was totally unplanned; for others, it built on pre-existing thoughts about quitting which had not yet been acted upon. Smokers interested in quitting were comfortable about approaching the MSSS, whilst acknowledging that they did not feel pressured to register with the service. The drop-in format of the MSSS was found to be more appealing than making an appointment. In addition, several participants articulated that they may not have utilised other SSS had they not come across the MSSS.

**Conclusions:**

A MSSS may be an effective way to prompt quit attempts for smokers not planning to quit and also reach smokers who would not engage with SSS.

## Background

In the United Kingdom (UK), the association between smoking prevalence and social disadvantage is well documented. Adults from socio-economically deprived groups and/or areas, as determined by level of education, housing tenure, social class and income level, are more likely to smoke and less likely to quit when compared to their more affluent counterparts [1]. Smoking prevalence has declined between 1980 and 1996 in the general population from 39% [2] to 28% respectively [3]. In recent years however, the decline has slowed down and prevalence was reported as being 22% in 2008/09 [3]. In contrast, differences according to socio-economic factors remain marked and unchanged [4], with 32% of men and 27% of women estimated to smoke in routine and manual occupations compared with 17% of men and 14% of women in managerial and professional groups [3]. Smoking therefore remains a significant contributor to health inequalities; for example, it is the main factor associated with higher death rates in the manual as compared with the non-manual occupation group [5]. Therefore, smoking is an important factor when attempting to understand reasons for those from less affluent groups experiencing poorer health outcomes and decreased life expectancy when compared with those from more affluent groups [6]. Hence, efforts to increase engagement of smokers from less affluent socio-economic groups are a priority for Public Health [7,8].

Whilst the provision and uptake of National Health Service (NHS) stop smoking services (SSS) has improved in recent years [9], only 8% of all smokers utilise them [3]. Typically, SSS involve smokers booking an appointment at clinics often held in health or community centres, where they meet with a trained advisor for one-to-one or group behavioural support and receive pharmacotherapy treatment [10,11]. The success of these services is undisputed [12], with chances of quitting increased by four-fold when compared with willpower alone [11]. A review of NHS SSS in general, summarises that some have been successful in attracting smokers from deprived areas and thus have worked towards addressing health inequalities [13]. However, similar to other health screening initiatives [14-16], the uptake of SSS by smokers from poorer socio-economic groups remains a challenge [13,17], due to barriers such as fear of being judged, fear of failure, lack of knowledge about the existence and nature of SSS and availability of pharmacotherapy [5]. Novel approaches to engage, recruit and support smokers from these groups are required [18-22]. For example, when considering breast cancer screening, services located at non-health facilities were perceived as more accessible than those at health facilities; however, reasons for this requires further research [14]. At present, strategies to recruit smokers from disadvantaged groups are limited, although there is some evidence that providing SSS in alternative settings such as workplaces could improve access [17]. The Roy Castle Fag Ends SSS in Liverpool (UK) is an example of a client-led approach that is flexible offering both one-to-one or group support where there is no waiting list, clients choose whether to make an appointment or drop-in, and they decide when to stop attending [23]. Considerable success has been attributed to the accessibility of the service with self-referral by drop-in accounting for 41% of total clients seen in 2005, an increase from 19% in 2001. In addition, 57% of clients were abstinent from smoking at 4 weeks (annual average over the period 2001 to 2005 [23]).

The need for more dynamic and flexible SSS is further supported by research suggesting that a notable proportion of quit attempts are unplanned and spontaneous [24,25]. The PRIME (plans, responses, impulses, motives, evaluations) theory of motivation [26] argues that smokers can be prompted to quit, without prior thought or planning [24,27]. Research also indicates that it is possible to support smokers with varying levels of motivation to quit [27]; thus questioning the usefulness of behaviour change models such as stages of change in the tobacco domain [28]. Making unsupported unplanned quit attempts does not appear to differ according to socio-economic groups and are often triggered by advice from a health care professional [29]. However, efforts to further understand spontaneous quit attempts have found that, for some, an element of planning was inherent [30,31]. This study explores the views of smokers who registered with a mobile, community-based SSS (MSSS) taken to socio-economically disadvantaged areas in Nottingham, over 4 weeks (September to October 2010); this formed part of a pilot study that was conducted prior to a main study of the MSSS's effectiveness.

## Methods

### The MSSS

**Figure 1:**
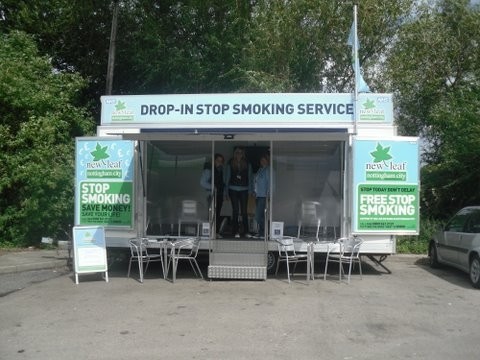
**Image of the MSSS**.

The MSSS was a drop-in service run in collaboration with Nottingham City's existing NHS SSS, New Leaf, using their branding. The service was run from an exhibition trailer (Figure 1) and was staffed by two trained stop smoking advisors and a support worker who was present outside the MSSS as a first point of contact and provider of information about the service. The advisors followed the same protocols as New Leaf, where clients received an initial consultation lasting approximately 30 minutes during which behavioural support and pharmacotherapy treatment (by delegated prescribing) were provided and clients were supported to either quit now or on an agreed date. The client was then encouraged to attend weekly follow-up consultations with an advisor for up to 12 weeks following their quit date, either at the MSSS (drop-in basis) or a fixed clinic location (appointment/drop-in; varies according to clinic so clients may have to wait if advisor busy with another client). During these sessions lasting approximately 15 minutes, the advisor provided further behavioural support, monitored carbon monoxide levels and arranged pharmacotherapy treatment (nicotine replacement therapy [NRT] given direct to the client), according to the needs of the client. Clients were informed that they could contact an advisor if support was required between follow-up (reactive telephone support). If clients failed to attend follow-up the advisors would attempt to contact the client via telephone on up to three occasions, and then a letter was sent via post. Thirteen locations in socio-economically disadvantaged areas, identified using the MOSAIC classification of Nottingham households [32,33] that could accommodate the trailer were selected; five supermarket/retail centre car parks, four leisure centre car parks, two industrial estates and two community/medical centre car parks. The MSSS visited ten of these locations on a single occasion and three on more than one occasion.

### Study design and participants

Individuals who registered with the MSSS were invited to take part in a telephone interview by the smoking cessation advisor at the end of the initial consultation. Of 151 smokers who had an initial consultation, 81 (53%) provided consent to be interviewed and were informed that a researcher would attempt to call within a few days. The interviewer aimed to conduct the interviews within a week of the initial consultation between September and November 2010; however, this varied between one and ten days. Eleven cases provided invalid contact details; the interviewer attempted to contact the remaining 70 individuals on at least four occasions and 40 (57%) were interviewed. After 40 interviews, data were saturated [34]; therefore no further attempts were made to contact the remaining 30 individuals. The age, gender and employment status distribution of those not interviewed was similar to the interviewed participants (mean age = 41 years and 42 years; male = 48% and 45%; employed = 70% and 63%).

Socio-demographics are presented in Table 1. At least one individual was interviewed from 12 of the 13 different MSSS locations; for the remaining location, only one individual consented to interview and did not respond to the four calls.

**Table 1:** Participant socio-demographics

*N *= 40	*n*	%
Age (years)*		

17-25	3	7.5

26-34	11	27.5

35-43	9	22.5

44-52	11	27.5

53+	6	15

Sex		

Female	23	57

Male	17	43

Ethnicity		

White	40	100

Employment Status		

Employed	23	57

Unemployed	8	23

Home carer	7	16

Full-time student	2	4

*Mean = 42.13 years (standard deviation = 11.28; range 17-64 years)

### Interviews

A semi-structured interview guide was developed to explore clients' views of the MSSS and covered suitability of locations, publicity surrounding the service, the usefulness of the support worker, views about the service, the appropriateness of the trailer, whether individuals had set quit dates, intentions to attend follow-up and ways the MSSS could be improved. Participants were informed that data would be anonymised, treated confidentially and that they were free to withdraw at any point during the interview, if they so wished. Interviews were conducted in a private room at Nottingham City Hospital (by M.B.) via telephone, lasted 16 minutes on average (ranged between 10 and 55 minutes) and were digitally audio-recorded.

### Data analysis

Interviews were transcribed verbatim by an external specialist transcription company. Following receipt of the transcripts, the interviewer removed any identifiers and ensured transcripts were accurate. Participants were assigned a unique code (e.g. 169F27) that identified date of recruitment (169; 16th September), their gender and age (F; Female, 27 years). Transcripts were analysed using thematic analysis [35]. This involved the interviewer reviewing each transcript separately. Transcripts were read several times and initial ideas were noted by hand, prior to using NVivo 8 (QSR International Ltd, Melbourne, Australia) as a data management tool. This guided the development of preliminary codes and an appropriate codebook. The resulting codes were grouped into potentially relevant themes and were discussed between the interviewer and a second researcher (L.L.J.). The themes were then reviewed to check if extracts represented them appropriately. This allowed clarification regarding the specific nature of each theme, leading to the development of names and descriptions for each core theme. Following agreement of the themes, extracts were taken from the transcripts to exemplify each theme and reflect the overall accounts reported by the participants.

### Ethical approval

A favourable opinion for the study was given by Leicestershire, Northamptonshire and Rutland Research Ethics Committee 2 (10/H0402/35) and the Research and Development department at Nottinghamshire County Primary Care Trust.

## Results

The analysis revealed six core themes, of which three were practically oriented and only relevant for planning the main trial so are not reported here (publicity and signage, New Leaf service and regularity of the MSSS). Data presented in this paper relates to the remaining three themes: the MSSS triggering quit attempts, first impressions and approaching the MSSS and accessibility and appropriateness of the MSSS.

### MSSS triggering quit attempts

**Figure 2:**
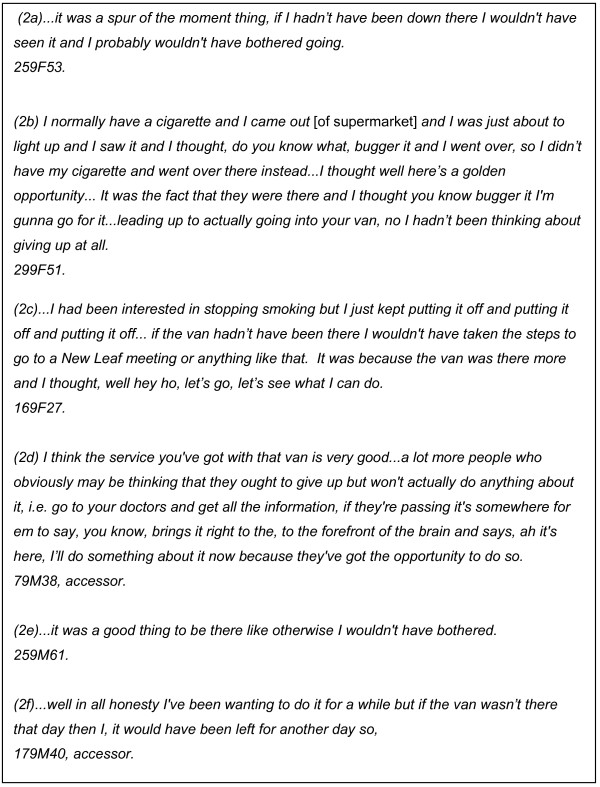
**MSSS triggering unplanned and planned quit attempts**.

The MSSS seemed to trigger quit attempts for a number of participants: for some of these participants the quit attempt was totally unplanned; for others it built on pre-existing thoughts about quitting. It was apparent that the MSSS triggered truly unplanned quit attempts in some cases, and for these individuals prior thoughts about quitting appeared absent (Figure 2). These individuals indicated that after seeing the MSSS, seemingly by chance, they decided almost instantly that they wished to cease smoking (2a, b). For others, it seemed that they had thought about quitting previously and that seeing the MSSS had served as a trigger and provided the ideal opportunity to engage with the service (2c). A few participants explained how they had been interested in stopping smoking for some time, but had put it off (2c). It was also suggested that the sight of the MSSS was likely to bring the issue of quitting to the forefront of individuals' minds (2d). Additionally, a couple of participants stated that the fact that the service was indeed mobile, creates a degree of uncertainty about whether such an opportunity would present itself again, thus individuals may be more likely to engage with a MSSS (4e, 2d). Several participants disclosed that they would not have utilised another form of SSS, had they not come across the MSSS, this was irrespective of whether quit attempts were unplanned or planned (2e, f).

### First impressions and approaching the MSSS

**Figure 3:**
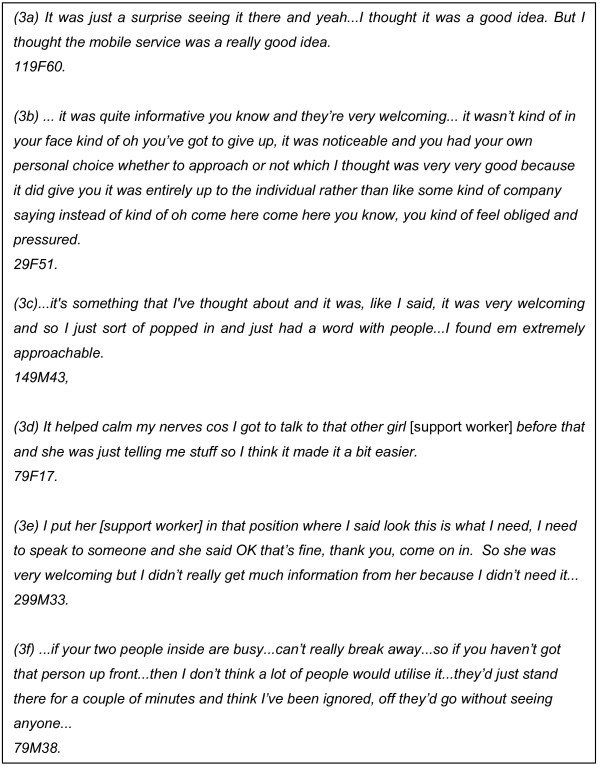
**First impressions and approaching the MSSS**.

Participants recalled positive first impressions of the MSSS and most were aware that it was a SSS. Participants described the MSSS as convenient, inviting and informative (Figure 3). Several participants were surprised to come across the MSSS, but felt the service was a good idea (3a). Individuals recognised that it was up to them to approach the MSSS if they were comfortable in doing so, and this personal choice seemed to be important (3b). Reasons for this were associated with the MSSS being perceived as approachable, perhaps because a support worker was present outside the trailer (3c). The majority of participants stated that they had spoken to the support worker and felt that this initial contact was important (3c, d). The support worker appeared to calm individuals who felt nervous about approaching the MSSS and who were perhaps unaware of the precise nature of the service being offered, particularly what a consultation would entail. Even though a small minority did not speak with the support worker or viewed this role as less important (3e), they recognised this role could be important for others, particularly when the stop smoking advisors may be occupied with clients (3f). Additionally, a couple of participants mentioned that speaking to the support worker was helpful as they were able to receive information without feeling pressurised or committed to register with the service (3b).

### Appropriateness and accessibility of the MSSS

**Figure 4:**
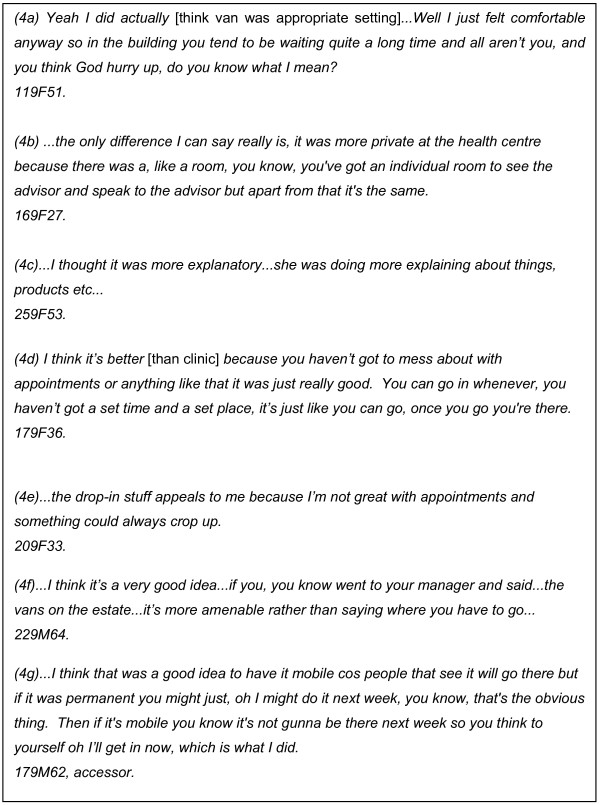
**Appropriateness and accessibility of the MSSS**.

The mobile unit was deemed to be an appropriate setting for the delivery of a SSS (Figure 4). Some participants had accessed SSS previously, but in more traditional settings, such as at health clinics. A proportion of these individuals preferred the MSSS and found it more appealing than having to visit a clinic-based setting, mainly because they felt the setting was more approachable, comfortable and because some seemed to associate traditional clinics with having to wait (4a); with the only downside being that traditional settings offered more privacy (4b). Greater appeal came from the MSSS being perceived as more welcoming and that participants were provided with more information compared with their prior encounters with the same service received in a clinic (4c). However, another individual felt that although the consultation in the MSSS was quicker than a prior experience at a clinic, the service provided was similar. When accessing SSS previously, it was apparent that appointments may have served as a barrier and resulted in non attendance (4d, e). Therefore, the MSSS may have been perceived as more convenient, particularly for those who accessed the service near to their workplace and perhaps because they could drop-in during a break (4f). The drop-in format also seemed to suit several participants who attended follow-up at the MSSS, to discuss their progress with an advisor; suggesting that this format may also increase the likelihood of individuals attending follow-up, particularly for those who accessed the MSSS near to their workplace (4e, f). Finally, the notion of it being a mobile service led some participants to mention that 'spur of the moment' visits were increasingly likely, as they did not need to book appointments and/or were uncertain whether it would be there in the future (4d, e, g)

## Discussion

This study demonstrates that MSSS may be an effective way to reach smokers who may not normally engage with SSS. Although we did not explore the views of individuals who chose not to access the MSSS, our findings suggest that smokers who wanted to quit were comfortable about approaching the MSSS, whilst acknowledging that they did not feel obliged to register with the service. Great appeal was associated with the drop-in format over making an appointment, suggesting that future programs should be designed so that smokers perceive them as being flexible and accessible; although the precise details regarding what it is that makes one SSS seem more accessible than another needs to be studied further. The MSSS appeared to reach smokers who were perhaps less likely to utilise traditional SSS and who seemed to embark upon a quit attempt after coming across the service, apparently by chance. The MSSS therefore may go some way in addressing the limitations of previous attempts to improve access to SSS by these groups [17]. The MSSS may offer a more personalised approach that is flexible [5], whilst providing the same support offered by traditional SSS.

It emerged that for a proportion of participants, the MSSS appeared to trigger quit attempts. For some participants the quit attempt was unplanned; for others, it built on pre-existing thoughts about quitting which had not been acted upon, a finding that is supported in the literature [24,25,30]. Importantly, several participants disclosed that they would not have utilised traditional SSS had they not come across the MSSS. The drop-in format may have been particularly conducive for attracting this group of smokers, mainly because their quit attempts appeared to be less planned. This is further supported by the PRIME theory which proposes that smokers can be prompted to quit by creating a rule to not smoke at any given moment, without pre-planning [25,26] and thus even those in early motivational stages to quit could benefit from SSS [27]. Therefore, MSSS could be an effective way of prompting such quit attempts [26]; it may also help to improve our understanding of spontaneous quit attempts, and how to provide more effective support to these smokers. It is also important to consider the success of spontaneous quit attempts compared with more planned attempts, particularly when evaluating services such as a MSSS. Early research indicates that spontaneous quit attempts yield more successful outcomes than planned attempts [24,25]; however these studies have been argued to be flawed methodologically, due to their cross-sectional designs and failure to control for confounding factors such as recall bias. More recent research that adopts a longitudinal approach suggests that prior planning was unrelated to outcomes [31]. The advantage of the MSSS triggering spontaneous quit attempts is that support is available at the point the quit attempt is initiated; spontaneous quit attempts are often, by their very nature, unsupported [29,30], hence increasing the likelihood of failure. Further exploration of this issue will be possible during the main MSSS study period.

The MSSS format may also overcome smokers' concerns about approaching traditional SSS, particularly those operating within medical facilities [14]. Individuals who approach the MSSS are able to decipher important features that may help to put them at ease; for instance, the presence of a support worker, who provided information about the service. This role may have overcome the barrier of not knowing what to expect, previously cited as a reason for not accessing traditional SSS [5]. Individuals may have used visual information regarding the service to decide whether to access or not; accessing the MSSS through personal choice seemed important, where participants acknowledged that approaching the MSSS was their decision and that they did not feel obliged or pressured to register with the service. It is difficult to ascertain whether smokers perceive this as being the case with more traditional SSS, this is a matter for future research [14].

While it is recognized that this was not a representative sample of those attending the MSSS, it did include a high proportion of smokers that were unemployed or home carers (39%); thus this indicates that a MSSS is likely to engage smokers, previously identified as hard to reach [5,13,17]. Furthermore, implementing a MSSS in areas comprised of hard to reach groups may be an effective way to expose SSS to these individuals and this could result in increased engagement. However, further research is required to explore whether specific groups of smokers may find a mobile service more appealing than others, such as those from socially disadvantaged groups or spontaneous quitters; perhaps because this format may offer a more transparent service that is flexible resulting in more favourable perceptions of accessibility compared with more traditional settings held in health centres. Operating a drop-in format, rather than smokers arranging a fixed appointment, which is commonplace for traditional SSS, may have been particularly suitable for those accessing the MSSS; although it is noted that traditional SSS are beginning to offer drop-in services and these have been reported to be effective [23]. Moreover, appointments have previously been identified as a barrier for attendance in a number of health care settings, particularly because individuals report that they often have to wait for a substantial amount of time [36,37].

### Limitations

Whilst the findings are valuable, several limitations are acknowledged. Firstly, views around the MSSS were only explored in Nottingham, and the service is based on the City's single smoking cessation service, New Leaf. Hence, it is not known whether these smokers' views are representative of other SSS users across the UK. Secondly, even though over half of the individuals that consented were interviewed successfully, the sample was not ethnically diverse. Thirdly, whether the MSSS reached smokers from disadvantaged groups is difficult to ascertain because precise data regarding type of employment (manual/non manual), income and housing for those interviewed were unavailable. This is likely to be the case for any given MSSS and we argue that identifying locations deemed to target such groups may be the most effective way to increase engagement by these smokers. Finally, not having the views of smokers who initially expressed interest in the MSSS, but failed to register is also a weakness; however, consenting this group into the study proved challenging.

## Conclusions

In conclusion, MSSS appear to be an effective way of taking SSS directly to smokers, who may lack knowledge about, or be fearful of, existing services. Importantly, SSS which offer flexibility (e.g. drop-in) and go beyond more traditional settings may result in the service being perceived as more accessible by smokers, who may be more likely to engage as a result. Moreover, MSSS may be a useful way to engage smokers who may be less likely to engage with traditional SSS.

## Competing interests

The authors declare that they have no competing interests.

## Authors' contributions

All authors were involved in the conception of the project and development of the interview guide. MB conducted the interviews, analysed the data and drafted the manuscript. LLJ helped in analysis and aided draft of manuscript. AV, RL, AM and LLJ reviewed the manuscript. All authors read and approved the final manuscript.

## Pre-publication history

The pre-publication history for this paper can be accessed here:

http://www.biomedcentral.com/1471-2458/11/873/prepub
